# Regulatory Cross-Talk Links *Vibrio cholerae* Chromosome II Replication and Segregation

**DOI:** 10.1371/journal.pgen.1002189

**Published:** 2011-07-21

**Authors:** Yoshiharu Yamaichi, Matthew A. Gerding, Brigid M. Davis, Matthew K. Waldor

**Affiliations:** 1 Channing Laboratory, Brigham and Women's Hospital, Harvard Medical School, Boston, Massachusetts, United States of America; 2 Program in Biological and Biomedical Sciences, Graduate School of Arts and Sciences, Harvard Medical School, Boston, Massachusetts, United States of America; 3 Howard Hughes Medical Institute, Boston, Massachusetts, United States of America; Agency for Science, Technology, and Research, Singapore

## Abstract

There is little knowledge of factors and mechanisms for coordinating bacterial chromosome replication and segregation. Previous studies have revealed that genes (and their products) that surround the origin of replication (*oriCII*) of *Vibrio cholerae* chromosome II (chrII) are critical for controlling the replication and segregation of this chromosome. *rctB*, which flanks one side of *oriCII*, encodes a protein that initiates chrII replication; *rctA*, which flanks the other side of *oriCII*, inhibits *rctB* activity. The chrII *parAB2* operon, which is essential for chrII partitioning, is located immediately downstream of *rctA*. Here, we explored how *rctA* exerts negative control over chrII replication. Our observations suggest that RctB has at least two DNA binding domains—one for binding to *oriCII* and initiating replication and the other for binding to *rctA* and thereby inhibiting RctB's ability to initiate replication. Notably, the inhibitory effect of *rctA* could be alleviated by binding of ParB2 to a centromere-like *parS* site within *rctA*. Furthermore, by binding to *rctA*, ParB2 and RctB inversely regulate expression of the *parAB2* genes. Together, our findings suggest that fluctuations in binding of the partitioning protein ParB2 and the chrII initiator RctB to *rctA* underlie a regulatory network controlling both *oriCII* firing and the production of the essential chrII partitioning proteins. Thus, by binding both RctB and ParB2, *rctA* serves as a nexus for regulatory cross-talk coordinating chrII replication and segregation.

## Author Summary

There is scant knowledge of factors and mechanisms that link bacterial chromosome replication and segregation. We investigated the mechanisms that coordinate the replication and segregation of *Vibrio cholerae* chromosome II (chrII). Our findings suggest that control of *V. cholerae* chrII replication and segregation is linked by a regulatory circuit that involves sequences, including *parAB2*, *rctA*, and *rctB* (and their products), that flank this chromosome's origin of replication. The primary agent governing replication initiation is RctB; however, initiation can also be influenced by a previously characterized partitioning protein, ParB2, which we now show counteracts *rctA's* inhibitory effect upon chrII replication. Analogously, the autoregulatory *parAB2* locus is the primary determinant of chrII segregation; however, this process can also be influenced by RctB, which activates *parAB2* expression by binding to *rctA*. Thus, our findings suggest that the cross-talk between these two systems both prevents extreme fluctuations in protein and chromosome abundance and also enables coordination of chromosome replication and partitioning.

## Introduction

Efficient linkage of chromosome replication and chromosome segregation is necessary for all dividing cells. It is particularly important for maintaining balanced genetic content in organisms with more than a single chromosome, which includes a number of bacterial orders (e.g., *Vibrionaceae*, *Photobacteriaceae* [1]). However, there is relatively little knowledge of factors and mechanisms that link replication and segregation of bacterial chromosomes. For the gram-negative enteric pathogen *Vibrio cholerae*, whose genome is comprised of two circular chromosomes [2], distinct mechanisms that control the replication and segregation of each chromosome have been described, but no mechanisms for linking or coordinating these processes have been identified.

The two *V. cholerae* chromosomes have distinct initiator proteins that are specific for their target chromosomes. The initiator of chromosome I (chrI) replication is DnaA, a conserved AAA+ ATPase protein found in nearly all eubacteria [3]–[6]. *V. cholerae* DnaA binds and melts the origin of replication of chrI (*oriCI*) but not that of *oriCII*, the origin of replication of chromosome II (chrII) [7]. It is likely that regulation of DnaA-mediated initiation of *V. cholerae* chrI parallels DnaA-dependent control of replication initiation in *Escherichia coli* [6], [7].

**Figure 1:**
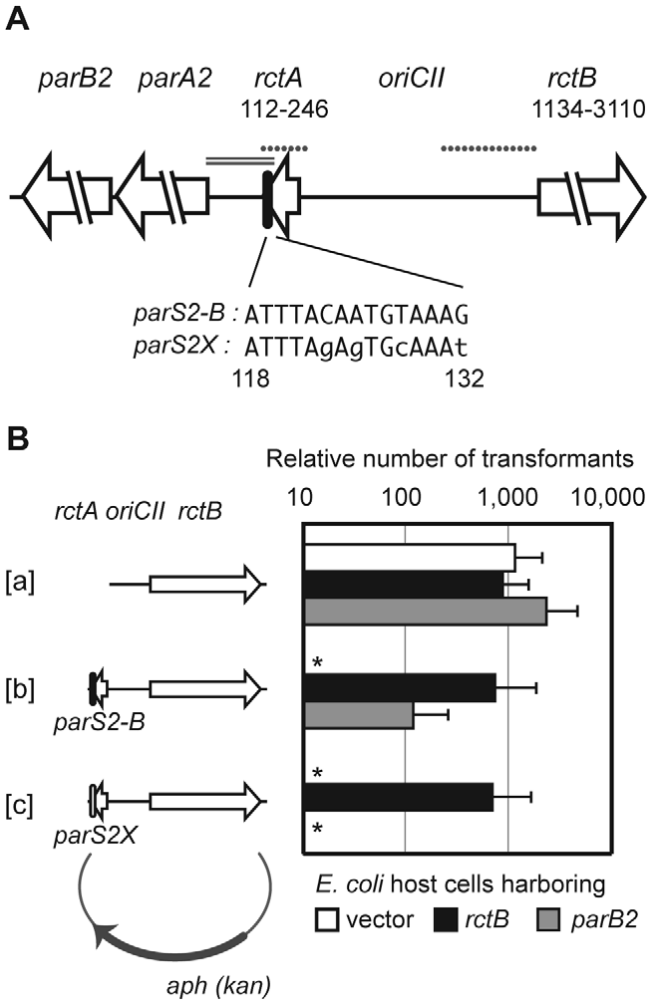
Interactions between RctB, *rctA*, ParB2 and *parS2* control *oriCII*-based replication. A) Schematic of *oriCII* region of *V. cholerae*. DNA fragments used as EMSA probes in Figure 3 are shown by dotted lines and the DNA fragment used in the transcription reporter assay in Figure 7 is shown by the double line. Native *parS2-B* and mutated *parS2X* sequences are also shown. Numbers correspond to genomic sequence data (NC_002506). B) Overexpression of RctB and ParB2 enable *oriCII*-based replication with origin fragments that include *rctA* and *parS2*. Self-ligated DNA fragments containing either *oriCII-rctB* [a], *rctA*-*oriCII-rctB* [b], or *rctA*(*parS2X*)-*oriCII-rctB* [c] were introduced into DH5α cells harboring control vector (pGZ119EH) (open bars), or *rctB* (pYB285) (closed bars) or *parB2* (pYB273) (gray bars) expression vectors. Mean and standard deviation of 5 independent experiments are shown. *No transformants obtained after overnight incubation in ≥3 experiments.

The initiator of chrII replication is RctB, a protein that is encoded near *oriCII* and conserved among, but restricted to, the *Vibrionaceae/Photobacteriaceae* (Figure 1A) [3]. RctB specifically binds and opens *oriCII* DNA in vitro, and its overexpression in *V. cholerae* leads to overinitiation of chrII but not chrI [4], [7]. RctB can bind and hydrolyze ATP, despite a lack of known ATP binding motifs; however, unlike other ATPase initiator proteins, the ATP-bound form of RctB is inactive for *oriCII* replication [7]. RctB activity is also negatively regulated by *rctA*, a neighboring gene [8]. Although *rctA* is transcribed [3] and was originally annotated as an ORF [2], it does not seem to encode a functional protein; instead at least one role of *rctA* appears to be as a DNA site for binding RctB, perhaps thereby titrating the initiator from *oriCII* [8]. Overall, the regulation of RctB activity and chrII replication initiation, which can be modulated by the factors noted above, by transcription within the *oriCII* region [8], and by additional proteins such as Dam and SeqA [3], [9], is complex and incompletely understood.

Although distinct proteins govern initiation of chrI and chrII replication, the replication of the two *V. cholerae* chromosomes is thought to be coordinated with the cell cycle, which should facilitate maintenance of genomic balance [10]–[12]. Genomic integrity is also promoted by chromosome-specific *par* systems, which have been implicated in the subcellular localization and/or partitioning of the respective *oriC* regions of each chromosome [13]–[16]. These systems consist of ParA ATPases, DNA-binding ParB proteins, and cis-acting ParB binding sites, *parS* ([17], [18] for review). The two *V. cholerae* ParB proteins (ParB1 and ParB2, encoded on chrI and chrII, respectively) recognize distinct *parS* sequences (*parS1* and *parS2*, respectively) [15]. While the nucleotide sequence of *parS1* is identical to the ‘universal’ *parS* sequence originally described in *Bacillus subtilis* [19], the nucleotide sequence of *parS2* is restricted to vibrio and photobacteria species [15], [20]. All but one of *V. cholerae's* 10 consensus *parS2* sites lie within chrII, and most of them are located proximal to *oriCII*. Interestingly, one of the *parS2* sites, designated *parS2-B*, is located within *rctA*, suggesting the possibility that this site could provide a basis for coordination of the control of chrII replication and segregation. Individual *parS2* sites are not essential for *V. cholerae* viability ([15] and data not shown); however, deletion of the chrII *parAB2* locus results in loss of chrII and cell death [16].

Here we explore how RctB interacts with *rctA* and how *rctA* negatively regulates chrII replication. Our observations suggest that RctB has at least two DNA binding domains - one for binding to *oriCII* and the other for binding to *rctA*. RctB lacking its C-terminus fails to bind *rctA* in vitro and its replicative activity is not inhibited by *rctA* in vivo. Notably, the inhibitory effect of *rctA* on RctB could also be alleviated by binding of ParB2 to the *parS2* site within *rctA*. Furthermore, ParB2 and RctB binding to *rctA* inversely alter the expression of the *parAB2* genes. Together, our findings suggest that fluctuations in binding of the partitioning protein ParB2 and the chrII initiator RctB to *rctA* underlie a regulatory network controlling both *oriCII* firing as well the production of the essential partitioning proteins ParA2 and ParB2. Thus, by binding both RctB and ParB2, *rctA* serves as a nexus for regulatory cross–talk coordinating chrII replication and segregation.

## Results

### A screen for factors enabling replication of an *oriCII*-based plasmid containing *rctA*

Previous studies have established that plasmids harboring *oriCII* (defined as the region between *rctA* and *rctB* ([3]; see Figure 1) as the sole origin of replication can replicate in *E. coli* as long as RctB is present, and that such replication can be inhibited by the presence of *rctA* either in cis or in trans [8], [21]. We utilized a similar approach to further dissect the molecular basis of *rctA*'s inhibition of *oriCII*-based replication. The replication capacity of plasmids that included *rctB*, *oriCII*, and various additional linked sequences were assessed by their efficiency of transformation into heterologous (*E. coli*) host strains containing either a control vector or one that overexpressed RctB (Figure 1B). Using this system, we obtained transformants within 24 hrs of introducing a plasmid that lacked *rctA* (pYB289), regardless of whether RctB was overexpressed (Figure 1B[a]). In contrast, no transformants were detectable 24 hrs after introduction of a plasmid that contained *rctA* (pYB292) unless the RctB expression construct was also present (Figure 1B[b]), consistent with the suggestion that sequestration of RctB by *rctA* reduces its replicative activity [8].

**Figure 2:**
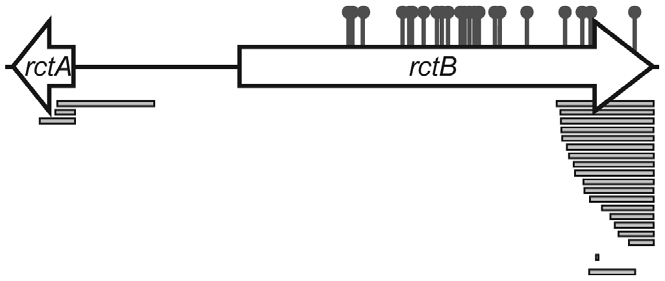
Map of locations of mutations that enabled replication of pYB292. Sites of amino acid substitutions are shown by the pins and deletions are shown as bars. More detailed information is presented in Table S1.

Notably, after ∼48 hrs, rare transformants were obtained with pYB292 even in the absence of RctB overexpression. Most of these colonies could be re-streaked, and plasmid DNA was recovered and sequenced from sixty five transformants. Sixty of these plasmids carried mutations that fell into one of three groups: 1) deletions of *rctA* (n = 6), 2) substitutions in the *rctB* sequence that result in amino acid substitutions in RctB (n = 34), and 3) substitutions or deletions in *rctB* that result in truncations of the carboxyl terminus of RctB (n = 20) (Figure 2 and Table S1). In general, strains carrying these plasmids grew at wild-type rates following the initial 24 hr lag in their detection, suggesting that the mutations within RctB did not impair its replicative capacity. Notably, none of the mutations mapped to the *oriCII* sequence per se, an observation that is consistent with the idea that an *rctA* transcript or protein does not act in trans on *oriCII*. In the remaining 5 cases, mutations were likely present in the host *E. coli* chromosome, since the purified *oriCII* plasmids (which did not harbor mutations) could be re-transformed into the DH5α strain they were isolated from (after plasmid curing) but not into a fresh isolate of DH5α.

### The C-terminus of RctB interacts with *rctA*

**Figure 3:**
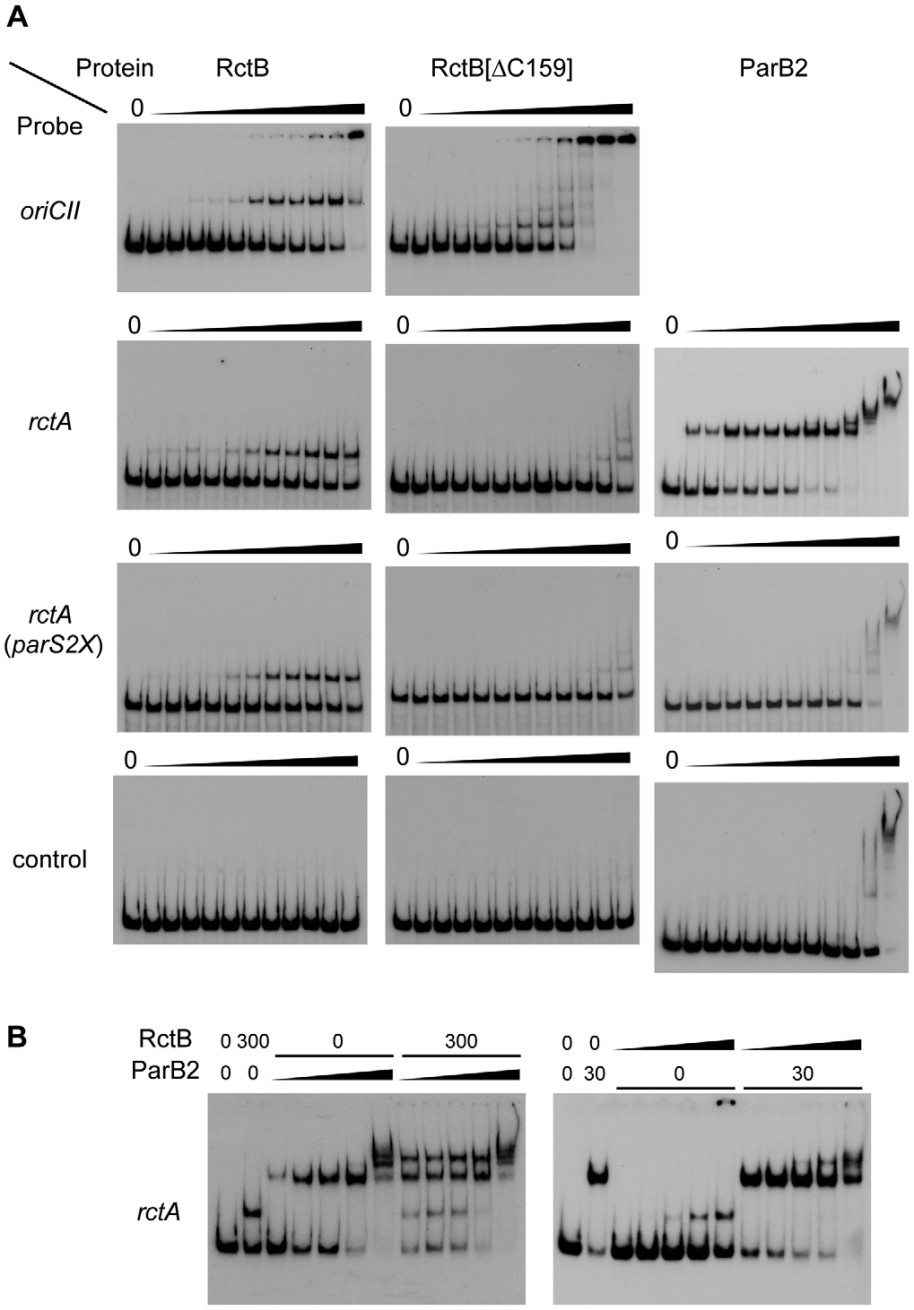
Binding of RctB and/or ParB2 to DNA fragments containing *rctA* and *parS2*. A) Binding of wild type or mutant RctB or ParB2 proteins to indicated DNA fragments. The amount of protein used in each lane was 0, 0.01, 0.03, 0.1, 0.3, 1, 3, 10, 31, 100, 316, 1000 ng, from left to right. B) Binding of RctB and ParB2 proteins to *rctA*. RctB and ParB2 were premixed and then added to the reaction tube. Amounts of proteins (ng) are indicated and the dilution series from left to right was 0.03, 0.3, 3, 30 and 300 ng. Note that similar banding patterns were observed in experiments where either RctB or ParB2 was added prior to the other protein (see Figure S1).

The prevalence (20 of 60 clones) of RctB truncations among pYB292 derivatives whose replication did not require RctB overexpression (Figure 2, Table S1) suggested that the C-terminal part of RctB might be required for its interaction with, and inactivation by, *rctA*. However, the normal replication of plasmids containing such truncations (predicted to remove at least 41, and at most 159, amino acids from the C-terminus of RctB) indicates that truncated RctB retains the capacity to melt *oriCII* and initiate chrII replication. Together, these observations raise the possibility that RctB has multiple sites and/or modes for interacting with DNA. To explore this hypothesis, we compared the binding of His-tagged full length RctB and RctB(Δ500–658), which lacks 159 amino acids of the protein's C-terminus (hereafter referred to as RctB[ΔC159]), to *oriCII* and *rctA*, using an electrophoretic mobility shift assay (EMSA). Both proteins readily bound to *oriCII*, and they appear to have a similar affinity for this sequence, although RctB[ΔC159] appears to have a greater tendency to form multimeric complexes on the DNA (Figure 3A). In contrast, while wild type RctB bound to the *rctA* probe, almost no binding of RctB[ΔC159] was detected (Figure 3A). Together, these observations suggest that RctB has at least two DNA binding domains; one, which binds *oriCII*, is contained within RctB[1–499] and can mediate *oriCII*-based replication, while the other, which binds *rctA*, is at least partially contained within, or dependent upon, sequences within RctB[500–658]. We were unable to demonstrate binding of RctB[500–658] to *rctA*, suggesting that additional regions of RctB likely also contribute to *rctA* binding. Thus, although some sequence similarity has been noted between potential RctB target sites within *oriCII* and *rctA* [8], our data raises the possibility that RctB actually recognizes two distinct sequences. Additionally, our data provides genetic and biochemical support for the hypothesis that RctB binding to *rctA* is the basis for *rctA*'s negative influence on *oriCII*-based replication.

### ParB2 binding to *parS2-B* alleviates the negative influence of *rctA* on *oriCII* replication

Similar to other chromosomal *parS* sites, most *parS2* sites are located proximal to *oriCII* on chrII [15], [21]. One of these (designated *parS2-B*) is found within the originally annotated *rctA* sequence (Figure 1A, ref. [15]). A *parS2* site is present at a similar position relative to *oriCII* in the genomes of multiple other vibrio species [15], despite an overall lack of conservation of the surrounding sequence. We hypothesized that ParB2 binding to this *parS2* site might influence binding of RctB to *rctA*, and perhaps thereby regulate *oriCII*-based replication. This possibility was investigated by measuring the effect of ParB2 on the efficiency with which various *oriCII*-related replicons could be transformed into *E. coli*. Overexpression of ParB2 had a minimal effect on the transformation of pYB289, consistent with the absence of *rctA/parS2B* within this construct (Figure 1B[a]). However, overexpression of ParB2 caused a dramatic increase in the efficiency with which the *rctA*-containing plasmid pYB292 could be introduced into *E. coli* (Figure 1B[b]). The effect of ParB2 expression was abolished when an alternate plasmid, pYB558, in which the *parS2-B* site was mutated to *parS2X* [15], was transformed instead (Figure 1B[c]). Transformants were still obtained with pYB558 when it was introduced into a strain that overexpressed RctB but not when it was introduced into a strain containing an empty vector, suggesting that the mutation in *parS2-B* did not interfere with binding of RctB, and thus that the two proteins do not recognize identical sequences (Figure 1B[b]). Data from the transformation assay was consistent with results from EMSAs, which revealed that ParB2 bound with high affinity to wild type *rctA* but not *rctA* containing *parS2X*, while RctB bound to both probes (Figure 3A). Overall, these data indicate that ParB2 binding to *parS2-B* can mask the negative effect of *rctA* upon replication of *oriCII*-based replicons.

### RctB and ParB2 can simultaneously bind *rctA*

**Figure 4:**
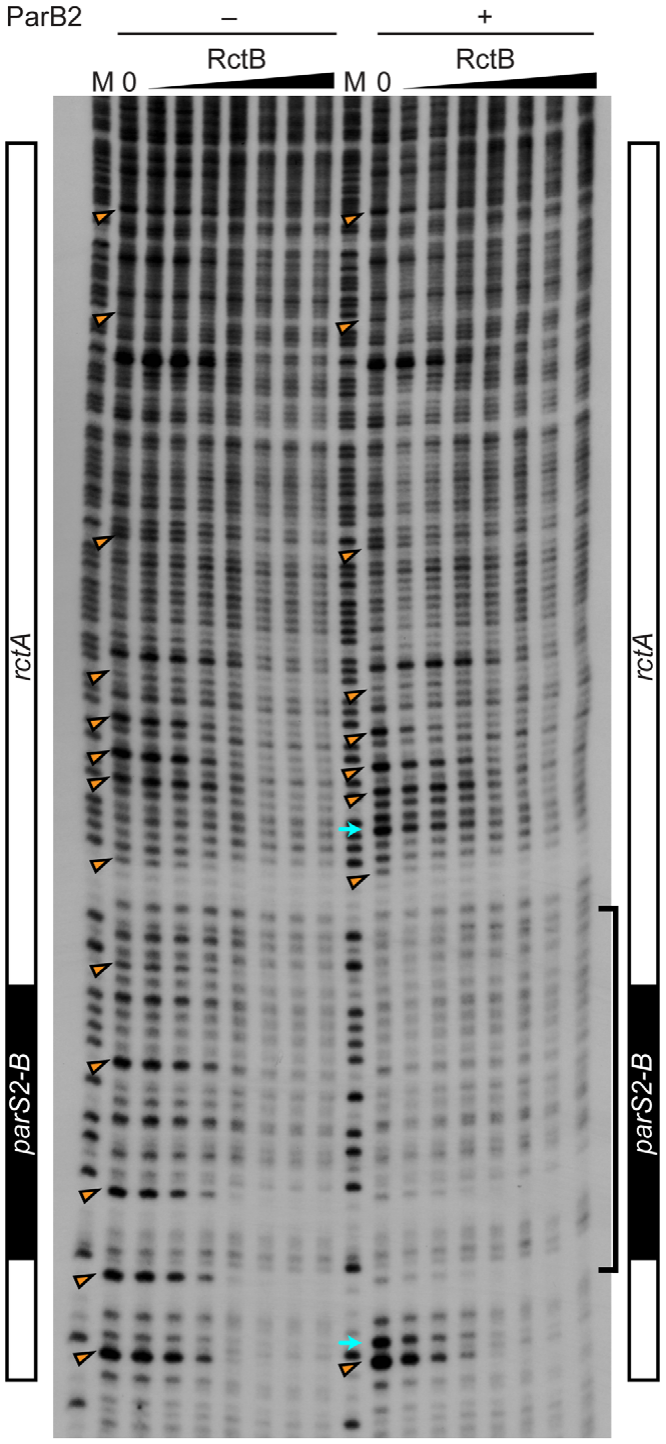
Protection of *rctA* from DNase I digestion in the presence of RctB or RctB and ParB2. The DNase I protection assay was performed with 0, 10, 20, 40, 80, 160, 320 and 640 ng RctB bound to a 5′-^32^P-labeled DNA probe containing *rctA* (including *parS2-B*, indicated at the side of the gel), in the absence or presence of ParB2 (100 ng). M denotes the G+A chemical sequencing ladder. The bracket indicates the ParB2 footprint. The arrowheads indicate nucleotides protected by RctB in multiple independent experiments. The arrows indicate hypersensitive sites.

The simplest explanation for increased transformation efficiency of pYB292 in the presence of overexpressed ParB2 is that binding of ParB2 to *rctA* interferes with binding of RctB to this site, and thereby makes more RctB available for replication initiation at *oriCII*. However, EMSA analyses did not provide direct support for this hypothesis. Instead, they indicate that RctB and ParB2 can bind simultaneously to *rctA* (Figure 3B and Figure S1). DNase I protection experiments confirmed that RctB can bind to *rctA*, but a specific region of binding was not observed (Figure 4). Instead, when ∼40–80 ng of RctB were added to the assay, several non-adjacent nucleotides that were distributed irregularly throughout the *rctA* sequence were protected from DNase I digestion (Figure 4, arrowheads). When higher amounts of RctB (∼160–640 ng) were added, the protection of individual bands became less pronounced and much of the fragment exhibited a degree of protection, including the *parS2-B* site. In contrast, ParB2 protected a ∼20 bp continuous stretch of DNA around the *parS2-B* site (Figure 4). Inclusion of both RctB and ParB2 in the DNAse I protection assays resulted in additive protection, consistent with simultaneous binding of both proteins to *rctA*. Additionally, DNAse I-hypersensitive sites (Figure 4, arrows) observed in the presence of ParB2 alone became protected upon inclusion of RctB in the reaction, suggesting that RctB can alter *rctA* structure even when ParB2 is bound. Collectively the EMSA and footprinting assays show that RctB and ParB2 can simultaneously bind to *rctA*. However, given the similar patterns of protection of the *parS2-B* region by the two proteins, it is difficult to ascertain whether ParB2 interferes with RctB's binding to this domain within *rctA*.

### Titration of ParB2 by ectopic *parS2* sites reduces *oriCII*-based replication

**Figure 5:**
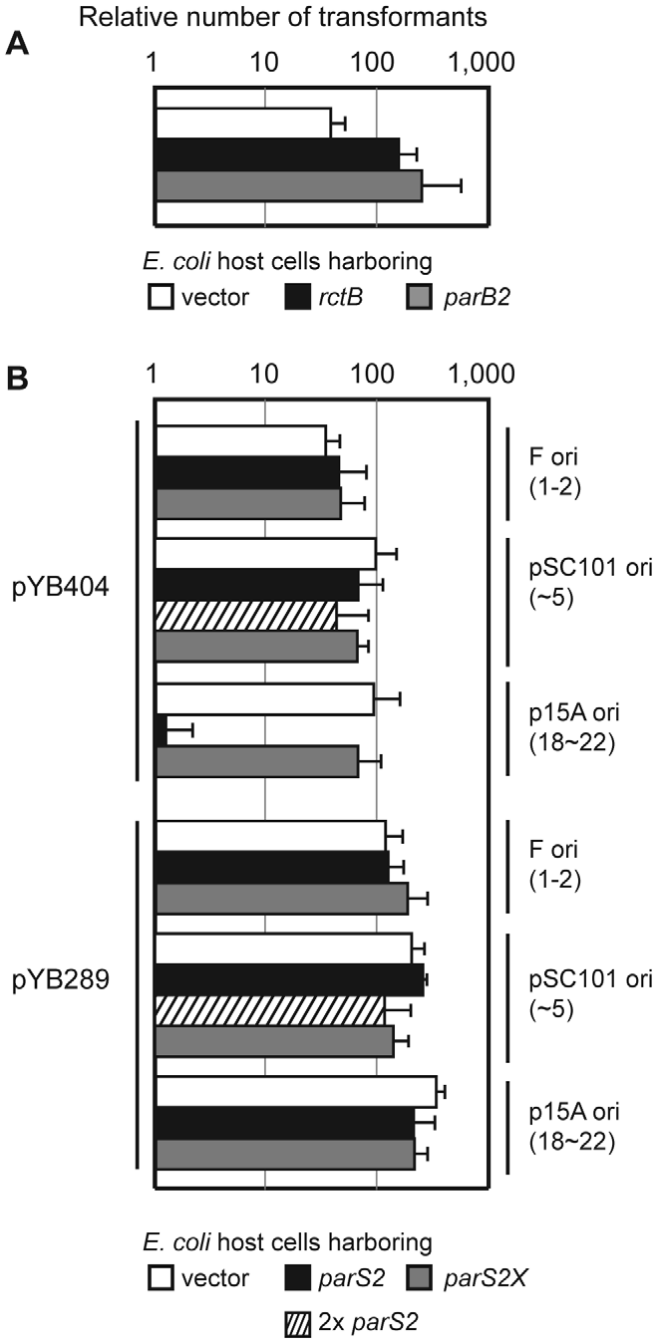
Titration of ParB2 by multicopy *parS2* prevents *oriCII* replication. A) Establishment of an *oriCII* plasmid containing *parAB2-rctA-oriCII-rctB* (pYB404) without overexpression of RctB or ParB2. B) pYB404 and pYB289 (*oriCII-rctB*) were introduced into DH5α cells harboring plasmids containing either *parS2* (closed bar), *parS2X* (gray bar), control (open bar), or two copies of *parS2* (hatched bar) with various replication origins (indicated on the right, with reported copy numbers shown in parentheses). Means and standard deviations from 3 independent experiments are shown.

In order to assess the roles of ParB2, *parS2* and *rctA* at more physiological levels in vivo, we generated an additional construct (pYB404) for the transformation assay that contained all 6 kb of DNA from *parB2* through *rctB* (Figure 1A), and thereby enabled expression of ParB2 from its endogenous promoter. In contrast to pYB292, pYB404 replicated in *E. coli* even without overexpression of RctB or ParB2, despite the presence of *rctA* in this construct (Figure 5A). Thus, ParB2 produced from its own promoter appears to be sufficient to overcome the negative effect of *rctA* on *oriCII*-mediated replication. However, overexpression of either RctB or ParB2 in the *E. coli* strain did increase the number of transformants obtained, perhaps because limiting amounts of these proteins are present when the plasmid is becoming established (Figure 5A).

We also assessed the influence on transformation efficiency of supplying additional copies of *parS2* from one of three plasmids with different origins of replication and copy numbers: F (1–2 copies/cell), pSC101 (∼5 copies/cell) and p15A (18∼22 copies/cell) [22]. The number of pYB404 transformants obtained was not altered if the ectopic *parS2* sequences were in the F-plasmid or even in pSC101 harboring two copies of *parS2* separated by 1.4 kb (pYB451, yielding ∼10 ectopic copies of *parS2* (Figure 5B). However, there was a marked decrease in the efficiency of pYB404 electroporation when the ectopic *parS2* sites were provided from the moderate-copy-number vector p15A (Figure 5B, p15A ori). In contrast, the *parS2X* sequences, which do not bind ParB2, did not alter pYB404 transformation efficiency when supplied from any of the vectors. (Figure 5B, gray bars). Neither wild type nor mutant *parS2* sequences altered the transformation efficiency of a vector (pYB289) that lacks *rctA*. Thus, the presence of 10 *parS2* sequences (notably, their level within the *V. cholerae* genome) is compatible with replication of a *oriCII*-based replicon containing *rctA/parS2B*. However, an increase to 20 copies (e.g., as should happen following *chrII* replication) interferes with *oriCII*-based replication, presumably because ParB2 is titrated away from the *parS2-B* site at the origin and thus can't counteract *rctA*-dependent repression. This finding suggests that ParB2 makes an important contribution to controlling replication as well as partitioning of *V. cholerae* chrII.

### ParB2-*parS2* and RctB-*rctA* interactions contribute to *oriCII* replication control in *V. cholerae*

To further explore the contributions of *rctA* and *parS2-B* to regulation of chrII replication, we constructed Δ*rctA V. cholerae* (YBB995) and *parS2-B::parS2X V. cholerae* (YBB999). These mutant strains did not have detectable growth defects compared to the wild type *V. cholerae* strain N16961 (Figure S2), indicating that *rctA* or *parS2-B* mediated control of *oriCII* is not critical for *V. cholerae* viability in rich media. However, quantitative PCR assays measuring the *oriCII* : *oriCI* ratio revealed that these mutations influence *oriCII* replication. The Δ*rctA* cells exhibited a higher *oriCII* : *oriCI* ratio compared to wild type cells (Table 1), in agreement with a previous report [23]. In contrast, the *parS2-B::parS2X* strain YBB999 had a modest but statistically significant (p = 0.001) reduction in the *oriCII* : *oriCI* ratio compared to the wild type (Table 1). Both of these results are consistent with our findings using the heterologous host and support the model that ParB2 binding to *parS2-B* inhibits the negative effect that *rctA* exerts on RctB-mediated *oriCII* replication.

**Table 1:** Ratio of *oriCII*/*oriCI* in *V. cholerae* strains.

Media	Strain	Genetype	*oriCII* / *oriCI*
LB*	N16961	wild type	1#
	YBB995	Δ*rctA*	1.53±0.06
	YBB999	*parS2-B::parS2X*	0.85±0.04
M9†	YBB703	wild type/vector	1#
	YBB682	wild type/*rctB^+^*	2.48±0.24
	YBB2003	Δ*rctA*/vector	1.31±0.06
	YBB2004	Δ*rctA*/*rctB^+^*	3.14±0.46
	YBB2005	*parS2-B::parS2X*/vector	0.94±0.13
	YBB2006	*parS2-B::parS2X*/*rctB^+^*	1.70±0.17

*Samples were obtained from mid-log phase cultures at an OD600 of ∼0.7.

†Samples were obtained from cultures grown in M9-Glucose supplemented with 100 µM IPTG for 2.5 hrs.

#Defined as 1.

**Figure 6:**
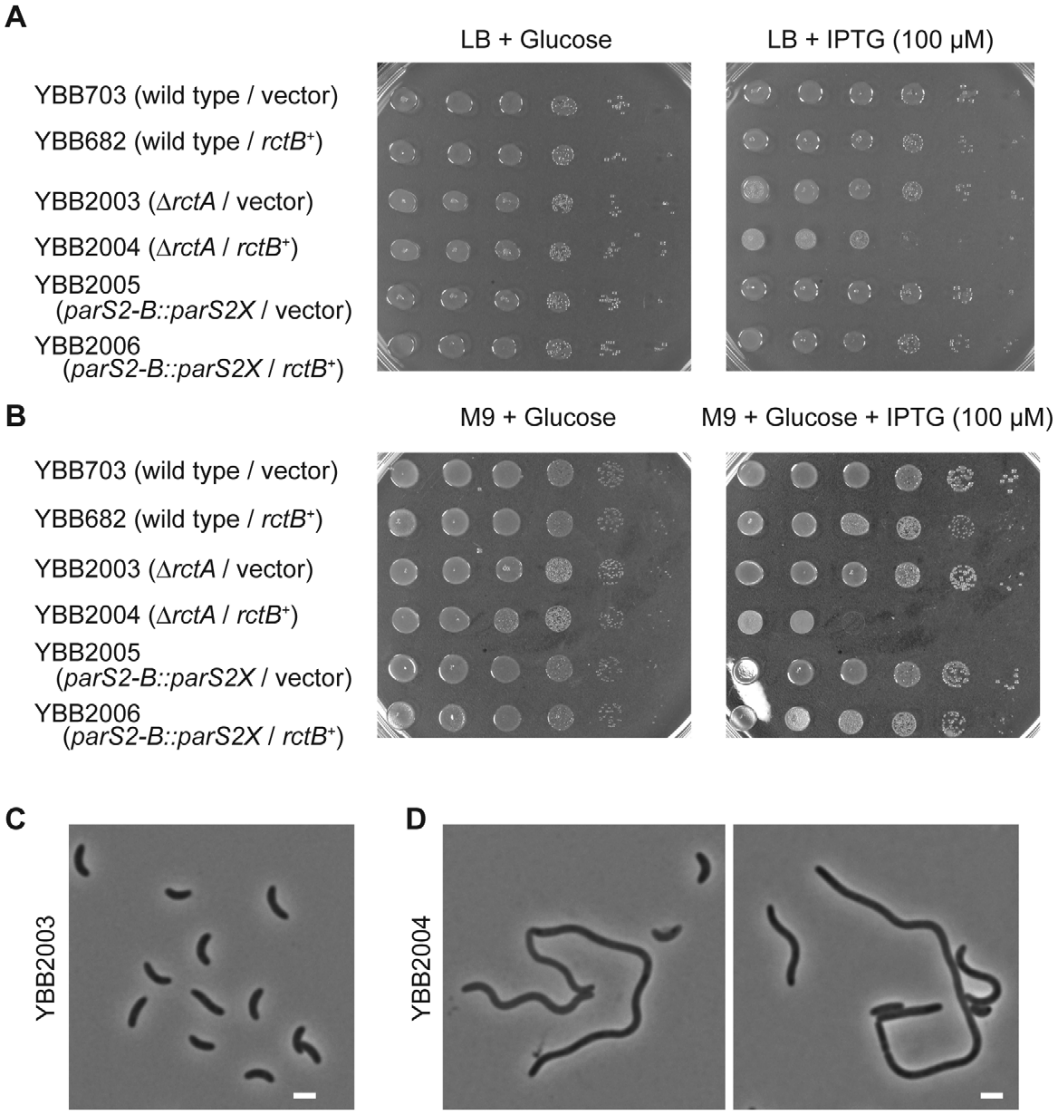
Overinitiation of *oriCII* can influence cell viability. A) and B) Plating efficiency of indicated strains on indicated media containing chrolamphenicol. C) and D) Phase-contrast images of YBB2003 (Δ*rctA*/vector) (C) and YBB2004 (Δ*rctA*/*rctB* ^+^) (D) after 4 hr of induction by IPTG. bar = 2 µm.

The causes and consequences of chrII overinitiation have not been thoroughly analyzed. However, previous work revealed that extreme overinitiation of *oriCII* mediated by the RctB mutant RctB[R269S] resulted in a block in *V. cholerae* cell division, manifest as cell elongation along with a marked decrease in viability [7]. In contrast, modest overinitiation of *oriCII* resulting from overexpression of wild type RctB had virtually no effect on cell viability or morphology (Figure 6A, 6C; see [4], [7], [23]). Similarly, the modest overinitiation of *oriCII* caused by the *rctA* deletion in YBB995 did not have a detectable affect on cell viability or morphology (Figure 6; [23]). However, deletion of *rctA* sensitized *V. cholerae* to the deleterious effects of RctB overexpression. In the Δ*rctA* background, RctB overexpression reduced cell viability, particularly in M9 media (Figure 6B), led to an increase in the *oriCII* : *oriCI* ratio (Table 1), and led to cell filamentation (Figure 6D). In contrast, in the *parS2-B::parS2X* background, RctB overexpression had little discernible influence on cell division or viability (Figure 6A, 6B), as might be expected given that the basal level of chrII replication initiation in this strain is even lower than in the wild type strain, whose viability was also unimpaired by RctB overexpression. Collectively, these data suggest that *V. cholerae* can adapt to some variability in RctB levels and availability, and that numerous regulatory processes are geared towards preventing the toxic effects of overinitiating replication of chromosome II.

### RctB and ParB2 control transcription of *parAB2*

**Figure 7:**
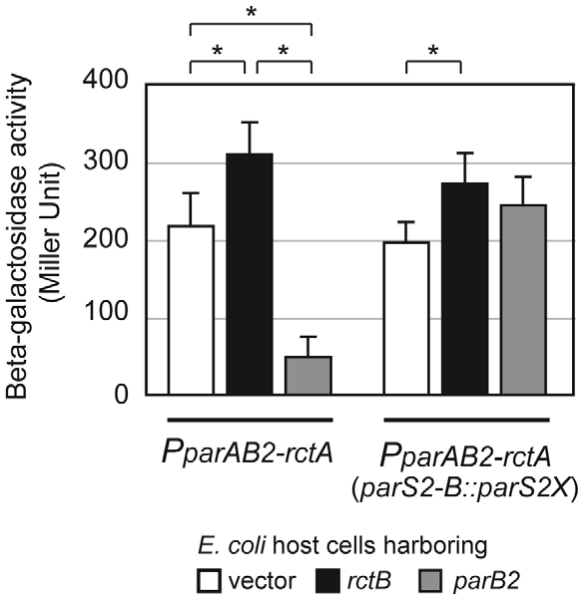
Transcriptional control of *parAB2* promoter by RctB and ParB2. β-galactosidase activities of *parAB2* promoter with or without an intact *parS2* were measured in the presence of a plasmid expressing RctB (pYB284) (black bars) or ParB2 (pSM922) (gray bars), as well as a vector control (pBAD33) (open bars). Averages and standard deviations are shown and * represents significant (p value<0.01) difference between the indicated groups.

Additional forms of cross-talk between RctB and the *parAB2* locus are evident from analyses of *oriCII*-region transcription, which revealed that binding of either ParB2 or RctB to *rctA* altered *parAB2* promoter activity. As has been observed for several additional *parAB* systems [24]–[26], ParB2 significantly decreased the expression of a *P_parAB2_* - *lacZ* fusion (more than 4-fold; Figure 7). This repression was abolished when *parS2-B* was mutated to *parS2X* (Figure 7), strongly suggesting that ParB2 binding to *parS2-B* is required for autorepression of the *parAB2* locus. In contrast, RctB modestly enhanced expression of *parAB2* (Figure 7, p = 0.0003), an effect that does not appear to depend on the *parS2-B* site in *rctA*. Thus, RctB binding to *rctA* may, despite initially limiting the amount of initiator protein available for replication initiation, ultimately promote replication, as such binding prompts expression of ParB2, which can counter repression of replication. Additionally, these results suggest that cross-talk between pathways controlling replication and partitioning is bidirectional, which is likely to enhance the coordination of these two critical processes.

## Discussion

Collectively, our observations suggest that control of *V. cholerae* chrII replication and segregation is linked by a regulatory circuit that involves ∼6 kb of sequence (and its products) that flank *oriCII* and includes *parAB2*, *rctA*, and *rctB*. The primary agent governing replication initiation is RctB; however, initiation can also be influenced by a previously characterized partitioning protein, ParB2, which we now show counteracts *rctA's* inhibitory effect upon chrII replication. Analogously, the autoregulatory *parAB2* locus is the primary determinant of chrII segregation; however, this process can also be influenced by RctB, which activates *parAB2* expression. It appears likely that the cross-talk between these two systems both prevents extreme fluctuations in protein and chromosome abundance, and enables coordination of chromosome replication and partitioning.

Binding of the chrII replication initiator RctB to the chrII origin and surrounding sequences appears to be more complex than was previously recognized. Our analyses indicate that RctB may in fact have multiple DNA binding modes/domains, which recognize distinct sequences. RctB lacking its C-terminus (as many as 159 amino acids) retained the capacity to bind to *oriCII* and to initiate replication at this site. However, both EMSA experiments and DNase I protection assays (Figure S3) revealed that RctB[ΔC159] is unable to bind to *rctA*. Residues outside of the C-terminal 159 amino acids are also likely to contribute to *rctA* binding, although they remain to be identified. The presence of distinct DNA binding domains within the N- and C- terminal parts of RctB introduces the possibility that a single RctB can simultaneously bind to *rctA* and *oriCII*. Additional studies are needed to assess whether the binding of RctB to these two sites introduces a bend in the DNA between them. Studies to assess whether the point mutations within *rctB* that enable a bypass of *rctA*-mediated replication inhibition do so by altering binding to *rctA* or instead alter other aspects of RctB's activity or its affinity for *oriCII* are also warranted.

To date, precise sequences targeted by RctB have not been identified; it has been speculated that this protein recognizes some short (11 and 12-mer) repeated sequences within the origin and surrounding sequences [3], [8]. Our footprinting analyses suggest that multiple RctB proteins are interacting with the DNA; however, the repeats do not seem to be the principal target of RctB's C-terminal DNA-binding domain, as many protected sites lie outside of the repeats. The distribution of sites within *rctA* that are protected from DNase I digestion by RctB is unusual, in that RctB appears to interact with multiple non-continuous bases throughout this sequence. One possible explanation for this result is that RctB binding alters the secondary structure of *rctA* DNA. Although the DNase I protection assays suggest that multiple RctB proteins interact with *rctA*, especially at high protein concentrations, EMSAs only revealed a single shifted band. The different assay conditions (e.g. the presence of competitor DNA in the EMSAs) may explain this apparent discrepancy. Additional analyses are needed to assess the binding sites for RctB in *oriCII*.

Our studies confirmed previous reports that *rctA* inhibits replication of *oriCII*-based replicons. The inhibitory effect of *rctA* can be overcome by overexpression of RctB (Figure 1B; [8]). Unexpectedly, our work revealed that *rctA*'s effect can also be mitigated by overexpression of ParB2, which recognizes a *parS2* site (*parS2-B*) within *rctA*. At least three non-mutually exclusive models can explain how ParB2 abolishes *rctA* inhibition of replication. One possibility is that ParB2 competes with RctB in binding to *rctA*, resulting in more free RctB that can interact with *oriCII*. However, both EMSA and DNase I protection assays demonstrated that ParB2 does not block all RctB binding within *rctA* in vitro; at most, only the subset of RctB binding sites in *parS2*-*B* are blocked by the presence of ParB2. However, these in vitro assays may not fully reflect binding dynamics in vivo in which binding to *rctA* maybe influenced by the adjacent *oriCII* site and by additional factors such as IHF. An alternative possibility is that RctB binding to *rctA* alters the secondary structure of the *oriCII* region in a manner that inhibits replication. ParB2 binding to *parS2-B* could counteract RctB-mediated remodeling of *oriCII*, thereby promoting replication. ParB2 might also alter the extent to which *rctA* is transcribed, which has also been shown to influence *rctA*'s effectiveness as a replication inhibitor [8]. It is unlikely that the effect of ParB2 upon replication is mediated by a direct interaction between this protein and RctB, as no such interaction was detected using a bacterial two hybrid system (Figure S4). Regardless of the mechanism by which it acts, it is clear that ParB2, previously described as a key agent mediating chrII segregation, also contributes to regulation of chrII replication, thereby enabling linkage of these cellular processes.

We hypothesize that the organization of this regulatory scheme is adapted to accommodate the cell cycle. As ParB2 accumulates, perhaps to amounts that are sufficient to enable chrII segregation, the repressive effects of *rctA* are relieved, and initiation of chrII replication ensues. Subsequently, ParB2 is re-distributed among the newly synthesized *parS2* sites, and its binding to *parS2-B* is reduced, enabling *rctA* to inactivate RctB, and thereby reducing the ability of RctB to initiate replication.

Cross-talk between chrII replication and partitioning is also evident at the level of transcription. The *parAB2* locus is autorepressed by *parB2*, as has been observed for other *parAB* loci [24]–[26]; in addition, we demonstrate that *parAB2* transcription is activated by RctB. The contrasting effects of these two regulators are likely to rebalance ParAB levels if their abundance becomes aberrantly elevated or reduced.

Undoubtedly, additional factors and mechanisms intersect with these regulatory circuits. For example, previous studies have revealed that RctB can repress its own transcription [27], [28] as well as the transcription of *rctA* [28]. Transcription of *rctA* has been reported to inhibit the negative influence of *rctA* on RctB [8]. Additional regulatory processes also contribute to control of replication initiation. For example, ATP binding inhibits RctB activity by decreasing its ability to bind *oriCII* [7] and the methylation status of *oriCII* also influences RctB binding to *oriCII* [9]. Given the centrality of chromosome replication and segregation to the perpetuation of the species, the existence of multiple and perhaps redundant mechanisms to increase the robustness of the control of these processes is expected. Consistent with this idea, we only observed significant impairment of *V. cholerae* growth when RctB was over-expressed in an *rctA* mutant, a condition that likely allows considerable overinitiation of chrII. Overinitiation also leads to growth impairment and cell filamentation in *E. coli* and *Caulobacter crescentus* [29]–[31].

Although coordinated control of chromosome replication and segregation makes sense to ensure proper chromosome inheritance to daughter cells, little mechanistic information linking these essential processes is available. Recent work by Murray and colleagues revealed that in *B. subtilis* the ParA ortholog Soj can inhibit or stimulate chromosome replication initiation via interactions with the initiator protein DnaA, while the ParB ortholog Spo0J inhibits initiation of chromosome replication by blocking Soj dimerization [32], [33]. A similar regulatory scheme was recently described for *V. cholerae* chrI; Chattoraj and colleagues reported that ParA1 stimulates chrI replication and ParB1 inhibits ParA1 [34]. However, ParA2 appears to govern chrII replication initiation via a distinct mechanism that does not require it to interact with the replication initiator RctB. In contrast to findings for Soj and ParA1, which interact with DnaA, we did not detect interaction between ParA2 and RctB using a bacterial two hybrid system. Our findings, along with previous reports, suggest that further exploration of the roles of Par systems in control of chromosome replication in diverse bacteria is warranted. Since chromosomal *par* genes are found in ∼70% of bacterial genomes [20], Par proteins and *parS* sites may commonly exert control over chromosome replication. Finally, it will be interesting to explore whether mechanisms exist to link the replication and/or segregation of the two chromosomes in *V. cholerae* and other bacteria with multiple chromosomes.

## Materials and Methods

### Plasmids and strains

Most of the plasmids used in this study are listed in Table 2. The sites and mutations present in the *rctA* containing *oriCII*-based plasmids (discussed in Figure 1B[b]) are shown in Table S1. The plasmids used for the bacterial two hybrid analysis are shown in Table S2.

**Table 2:** Plasmids used in this study.

Name	Description	Resistance†	Reference
pBAD33	p15A ori	Cm	[40]
pCB192-YY	pCB192 but no EcoRI site in 3′ of *lacZ*	Amp	[38], This study
pCVD442	vector for allelic exchange	Amp	[37]
pET-RctB	pET28b *rctB*	Km	[7]
pGZ119EH	ColD ori	Cm	[41]
pSM843	pET28b *parB2*	Km	[15]
pSM922	pBAD33 *parB2*	Cm	Sarah McLeod, This study
pWKS30	pSC101 ori	Amp	[42]
pXX705	F ori	Amp	[43]
pYB141	pXX705 *parS2-A*	Amp	This study
pYB190	pCRII	Km	[16]
pYB193	pBAD33 *parS2-A*	Cm	This study
pYB199	pKD4 MCS*	Amp, Km	This study
pYB216	pBAD33 *parS2X*	Cm	This study
pYB217	pXX705 *parS2X*	Amp	[15]
pYB264	pYB199 *rctA oriCII rctB*	Amp, Km	This study
pYB273	pGZ119EH *parB2*	Cm	This study
pYB276	pYB199 *oriCII rctB*	Amp, Km	This study
pYB285	pGZ119EH *rctB*	Cm	[7]
pYB289	*oriCII rctB*	Km	[7]
pYB292	*rctA oriCII rctB*	Km	This study
pYB294	pBAD33 *rctB*	Cm	This study
pYB355	pET28b *rctB*[ΔC159]	Km	This study
pYB379	pYB199 *rctA* (*parS2-B::parS2X*) *oriCII rctB*	Amp, Km	This study
pYB403	pYB199 *parB2 parA2 rctA oriCII rctB*	Amp, Km	This study
pYB404	*parB2 parA2 rctA oriCII rctB*	Amp, Km	This study
pYB405	pCRII *oriCII*	Km	This study
pYB406	pCRII *rctA*	Km	This study
pYB407	pCRII *rctA* (*parS2-B::parS2X*)	Km	This study
pYB432	pCVD442-based allelic exchange plasmid to construct Δ*rctA*	Amp	This study
pYB445	pCVD442-based allelic exchange plasmid to construct *parS2-B::parS2X*	Amp	This study
pYB447	pSC101 *parS2-A*	Amp	This study
pYB448	pSC101 *parS2X*	Amp	This study
pYB451	pSC101 *parS2-A parS2-A*	Amp	This study
pYB452	pCB192-YY *PparAB2-rctA lacZ*	Amp	This study
pYB453	pCB192-YY *PparAB2-rctA* (*parS2-B::parS2X*) *lacZ*	Amp	This study
pYB558	*rctA* (*parS2-B::parS2X*) *oriCII rctB*	Km	This study

*MCS; multi cloning site.

†Resistance; Amp, ampicillin; Cm, chloramphenicol; Km, kanamycin.

A two-step strategy for construction of *oriCII*-based plasmids was followed. First, different segments of DNA proximal to *oriCII* were amplified and cloned into pYB199, a derivative plasmid of pKD4 [35] which harbors the R6K origin and genes conferring resistance to ampicillin (*bla*) and kanamycin (*aph*). Second, the resulting plasmids were digested with XbaI and the fragment containing the *oriCII* region and *aph* was gel-purified, self-ligated and then electroporated into *E. coli* DH5α. Spontaneous suppressor mutants in the *rctA* containing *oriCII*-based plasmids (shown in Figure 2 and Table S1) were isolated by electroporating ∼100 ng of self-ligated DNA fragments into DH5α cells. Colonies that arose after an ∼48 hr incubation were re-streaked and then plasmid DNA was purified and sequenced. In addition, to confirm that mutation in the plasmid enabled establishment, each mutant plasmid was re-electroporated into DH5α. The mutations in *parS2-B* yielding *parS2X* (Figure 1A) were generated using the QuickChange XL Site Directed Mutagenesis Kit (Stratagene). To construct pYB141, pYB217, pYB447, and pYB448, 15-bp double stranded DNA fragments containing either *parS2-A* or *parS2X* were inserted into the EcoRI site of the vectors (pWKS30 and pXX705). Plasmids pYB193 and pYB216 were constructed in a similar fashion using the NheI and HindIII sites in the pBAD33 vector. To construct pYB451, the second copy of *parS2-A* was inserted at the AflII site of pYB447, which is ∼1.4 kb away from EcoRI site where the first copy of *parS2-A* was inserted. Plasmid pCB192-YY, a derivative of the transcriptional fusion vector pCB192 [36] in which the EcoRI site in the 3′ end of *lacZ* was removed by introduction of a silent mutation (GAATTC to GAATTT), was used to create transcriptional fusions to the *parAB2* promoter. The *parAB2* promoter region was amplified and cloned into the HindIII-EcoRI site of pCB192-YY. All the relevant DNA sequences of all plasmids used in this study were determined. The sequences of the oligonucleotides used in this study are listed in Dataset S1.

Mutations were introduced on to the *V. cholerae* chromosome (Δ*rctA*, and *parS2-B::parS2X*) via allele exchange using pCVD442-based plasmids as described [37]. *V. cholerae* strains used in this study are listed in Table 3.

**Table 3:** *V. cholerae* strains used in this study.

Name	Description	Source/Reference
N16961		[2]
YBB682	N16961/pYB285	[7]
YBB703	N16961/pGZ119EH	[7]
YBB995	N16961 Δ*rctA*	This study
YBB999	N16961 *parS2-B::parS2X*	This study
YBB2003	YBB995/pGZ119EH	This study
YBB2004	YBB995/pYB285	This study
YBB2005	YBB999/pGZ119EH	This study
YBB2006	YBB999/pYB285	This study

### Transformation efficiency experiments

DH5α cells harboring either pGZ119 (vector),or Isopropyl-β-D-1-thiogalactopyranoside (IPTG) inducible *rctB* or *parB2*, pYB285 or pYB273 respectively, were grown in LB broth containing 100 µM IPTG till mid-log phase to prepare electrocompetent cells. Similarly, DH5α cells harboring plasmid-borne *parS2* sequences or control plasmids were grown until mid-log phase to prepare chemical competent cells. 40 ng of self-ligated DNA or 10 ng of pYB404 DNA were introduced into the competent cells. As a control, 10 ng of plasmid pYB190 was also introduced into competent cells in each experiment. The number of colonies obtained in the pYB190 transformations were used to normalize transformation efficiencies. Means and standard deviations were derived from 3–5 independent experiments for all plasmids tested.

### Beta-galactosidase assays

Assays were performed in triplicate with log phase cultures as described previously [38]. Two tailed, two-sample equal t-tests were used to compare the results from 3 independent experiments (total 9 samples each) for the statistical analysis.

### Electrophoretic mobility shift assay (EMSA)

Wild type RctB-His6, RctB[ΔC159]-His6, and ParB2-His6 proteins were purified as previously described [15]. Sequences used for *oriCII*, *rctA*, and *rctA* (*parS2-B::parS2X*) (see Figure 1A) EMSA probes were initially cloned into the pCR-Blunt II-TOPO vector (Invitrogen), yielding pYB405, pYB406, and pYB407, respectively. pYB190, a pCR-Blunt II TOPO derivative containing an irrelevant 10 bp, was used to construct the negative control probe. To prepare radio-labeled probes, appropriate DNA regions were amplified from the plasmids with universal M13 forward and reverse primers [22], end labeled with [γ-^32^P] ATP with polynucleotide kinase (New England Biolabs), purified from 6% DNA retardation gels (Invitrogen), and ethanol precipitated. In the binding reactions, 5,000 cpm of probe DNA containing different concentrations of RctB and/or ParB2 in a reaction buffer of 20 mM Tris-Cl (pH 7.5), 1 mM EDTA, 150 mM NaCl, 12.5 µg/mL poly (dI-dC), and 0.1 mg/mL BSA were incubated for 10 min at room temperature. The reactions were then electrophoresed in a 6% DNA retardation gel in 0.5× TAE and visualized by autoradiography.

### DNase I foot print assay

DNase I footprint assays were performed as previously described with minor modifications [39]. The *rctA* probe was made by PCR using 5′-^32^P-radiolabeled rctA 5′-FP (CGTTTAAATAACCCACATATTCTTCGATAAGG) and rctA 3′-FP (ATACCTATTCGCTGGAGGAAAGATAGG) primers on a plasmid encoding *parAB2-rctA-oriCII-rctB* (pYB403). The probe was purified from 6% DNA retardation gels, eluted, and ethanol precipitated. 1,200,000 cpm of probe was incubated with different amounts of RctB without and with 100 ng of ParB2 in 20 µL of 20 mM Tris-Cl pH 8.0, 125 mM NaCl, 1 mM DTT for 10 min at room temperature. 0.1 U of DNase I (Applied Biosystems) was added to each reaction and incubated at room temperature for 30 sec. The digestions were quenched by the addition of 6 µL of 660 mM Tris-Cl pH 9.5, 66 mM EDTA, 3.3% SDS and placed on ice. Samples were ethanol precipitated, resuspended in recrystalized formamide, and 20,000 cpm of each was run on an 8% polyacrylamide gel with 8 M urea (National Diagnostics) in 1× TBE. The gels were then dried and visualized by autoradiography.

### Quantitative PCR assay

Genomic DNA was prepared from each strain by phenol-chloroform extraction followed by ethanol precipitation. The genomic DNA was then digested with PstI and 10 pg was used for each quantitative PCR (qPCR) reaction. Genomic DNA from an N16961 stationary culture was used to generate the standard curve. qPCR was performed with the StepOnePlus Real-Time PCR system (Applied Biosciences) using SYBR Green Master mix (Applied Biosciences) according to the manufacturer's protocol. The primer pairs used for *oriCI* and *oriCII* were described previously [4]. Each qPCR run was done in triplicate and the ratio was calculated from three independent experiments.

### Growth curves and microscopy

*V. cholerae* cells harboring a plasmid borne copy of an IPTG-inducible *rctB* or a control vector were grown in either LB or M9-Glucose media containing 100 µM IPTG at starting OD600 of 0.003. Subsequently, OD600 and CFU were monitored hourly. Growth curves shown in Figure S2 are representative of at least three independent experiments. A small aliquot of cells was removed at 4 hr, fixed with 3% paraformaldehyde, and then examined with 100× alpha-plan lens on a Zeiss Axioplan 2 microscope.

## Supporting Information

Dataset S1 List of oligonucleotides used in this study.
(XLS)

Figure S1 Binding of RctB and ParB2 proteins to *rctA*. A) and C), RctB was added to the reaction tube 8 min prior to addition of ParB2. B) and D), ParB2 was added to the reaction tube 8 min prior to addition of RctB. Amount of proteins in titration was 0.03, 0.3, 3, 30 and 300 ng, from left to right.
(TIF)

Figure S2 Growth curves of *V. cholerae* strains. OD600 nm (A) and Colony forming units (CFU) (B) of *V. cholerae* N16961 (gray circles), YBB995 (Δ*rctA*; open triangles) and YBB999 (*parS2-B::parS2X*; closed squares) cells grown in LB media at indicated time points are shown.
(TIF)

Figure S3 Protection of *rctA* from DNase I digestion by RctB[ΔC159]. The DNase I protection assay was performed with 0, 10, 20, 40, 80, 160, 320 or 640 ng RctB[ΔC159] bound to a 5′-^32^P-labeled DNA containing *rctA* (including *parS2-B*, indicated at the side of thegel). M denotes the G+A chemical sequencing ladder.
(TIF)

Figure S4 Interactions between RctB and ParB2. A pair of plasmids that express RctB or ParB2 fused to the T18 and T25 subunits of adenylate cyclase was simultaneously introduced into *E. coli* BTH101. After transformation, 2 µL of cells were spotted onto LB plates containing ampicillin (100 µg/mL), kanamycin (50 µg/mL), IPTG (100 µM), and bromo-chloro-indolyl-galactopyranoside (X-gal, 60 µg/mL) and incubated overnight at 30°C.
(TIF)

Table S1 List of mutant *oriCII* plasmids.
(DOC)

Table S2 List of plasmids used for bacterial two hybrid assay.
(DOC)
