# Supplementary figures and images for: Regulatory Cross-Talk Links Vibrio cholerae Chromosome II Replication and Segregation
Source: PLoS Genet. 2011 Jul 21;7(7):e1002189. doi: 10.1371/journal.pgen.1002189 (PMC3141006; doi:10.1371/journal.pgen.1002189)

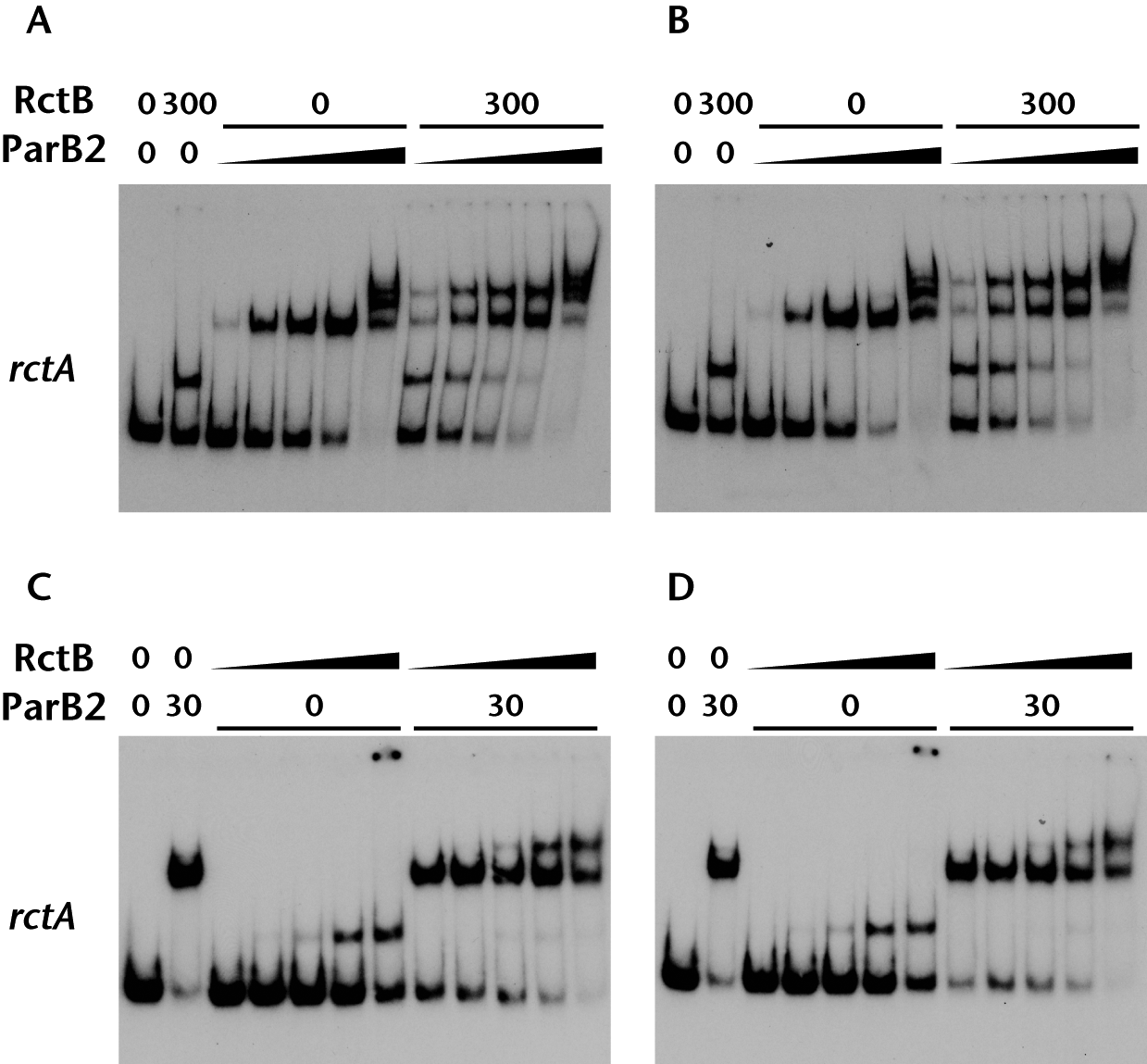

Supplement: Figure S1 — Binding of RctB and ParB2 proteins to rctA. A) and C), RctB was added to the reaction tube 8 min prior to addition of ParB2. B) and D), ParB2 was added to the reaction tube 8 min prior to addition of RctB. Amount of proteins in titration was 0.03, 0.3, 3, 30 and 300 ng, from left to right. (TIF) [file pgen.1002189.s002.tif]

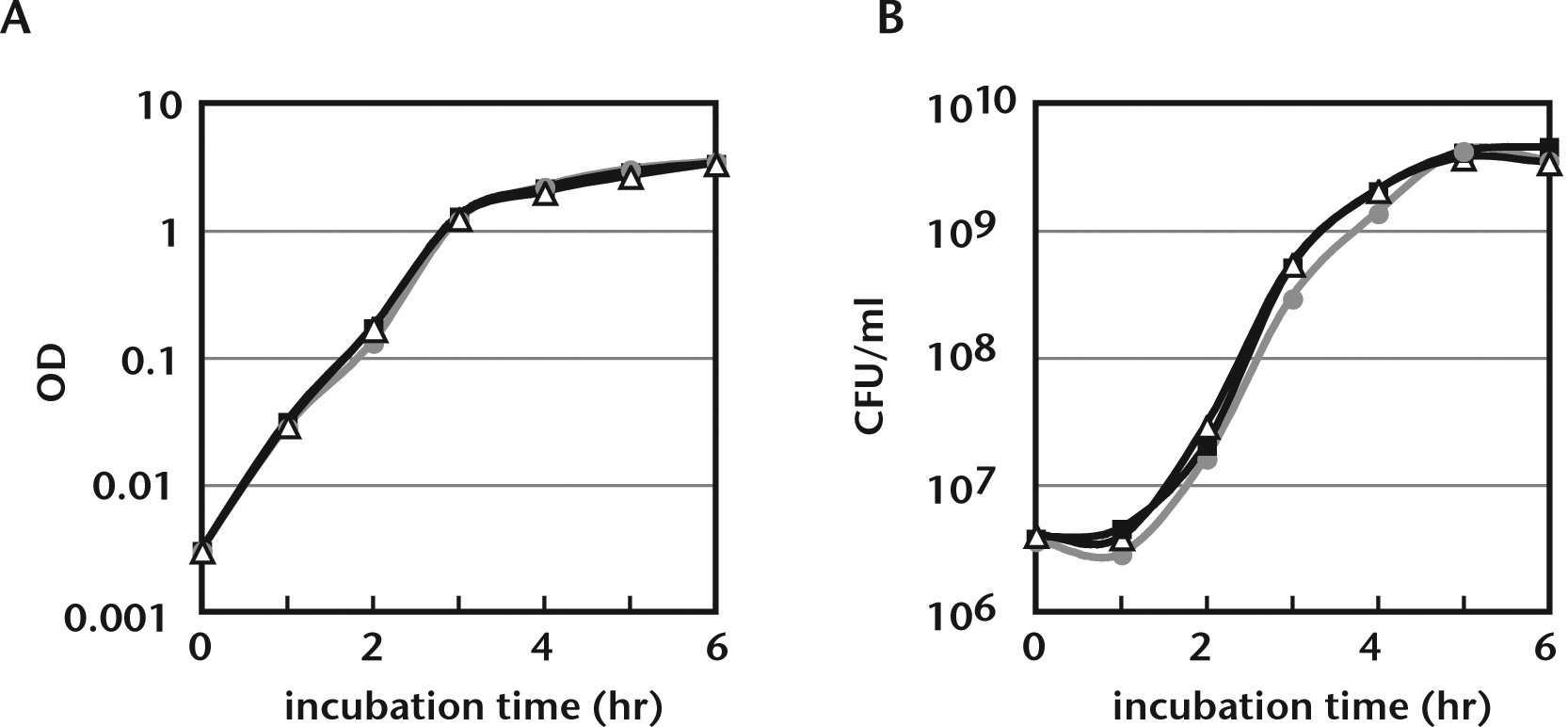

Supplement: Figure S2 — Growth curves of V. cholerae strains. OD600 nm (A) and Colony forming units (CFU) (B) of V. cholerae N16961 (gray circles), YBB995 (ΔrctA; open triangles) and YBB999 (parS2-B::parS2X; closed squares) cells grown in LB media at indicated time points are shown. (TIF) [file pgen.1002189.s003.tif]

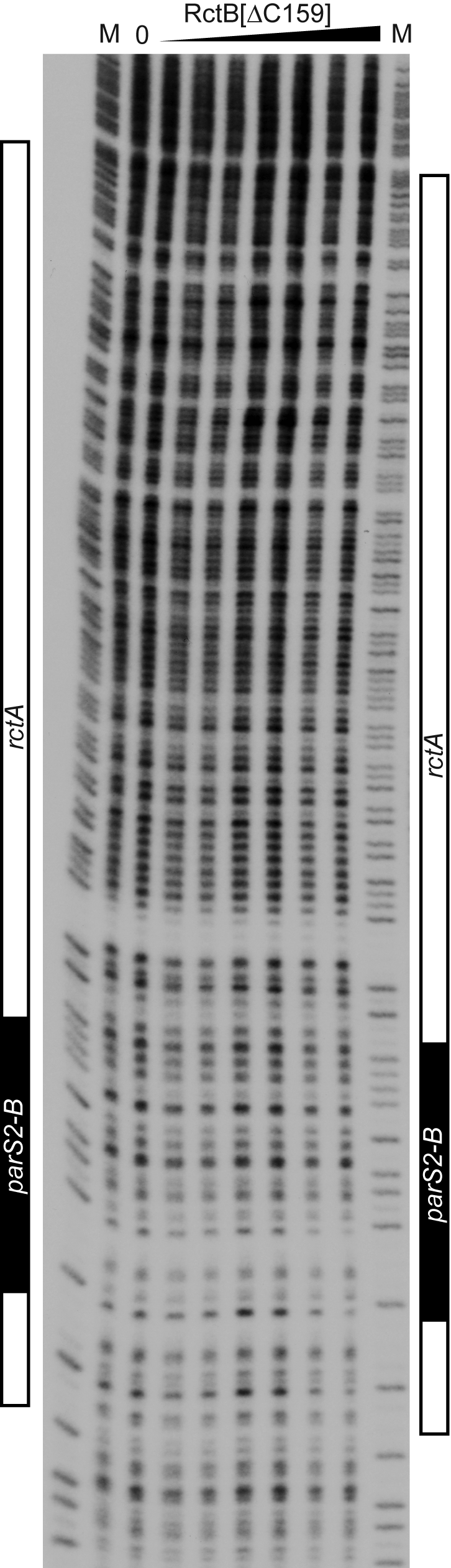

Supplement: Figure S3 — Protection of rctA from DNase I digestion by RctB[ΔC159]. The DNase I protection assay was performed with 0, 10, 20, 40, 80, 160, 320 or 640 ng RctB[ΔC159] bound to a 5′-32P-labeled DNA containing rctA (including parS2-B, indicated at the side of thegel). M denotes the G+A chemical sequencing ladder. (TIF) [file pgen.1002189.s004.tif]

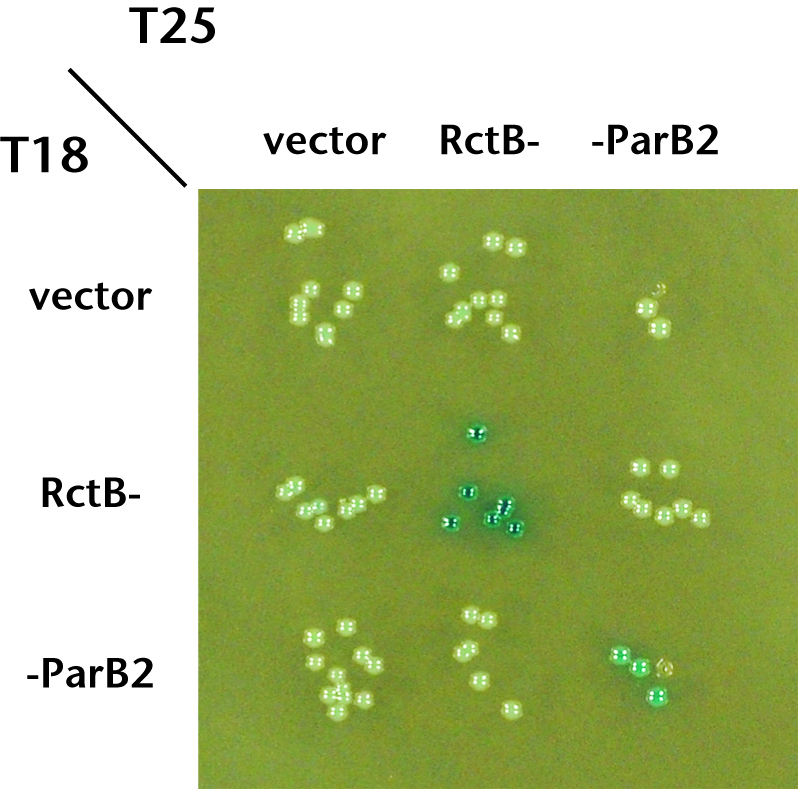

Supplement: Figure S4 — Interactions between RctB and ParB2. A pair of plasmids that express RctB or ParB2 fused to the T18 and T25 subunits of adenylate cyclase was simultaneously introduced into E. coli BTH101. After transformation, 2 µL of cells were spotted onto LB plates containing ampicillin (100 µg/mL), kanamycin (50 µg/mL), IPTG (100 µM), and bromo-chloro-indolyl-galactopyranoside (X-gal, 60 µg/mL) and incubated overnight at 30°C. (TIF) [file pgen.1002189.s005.tif]
