# Supplementary material for: Regulatory Cross-Talk Links Vibrio cholerae Chromosome II Replication and Segregation
Source: PLoS Genet. 2011 Jul 21;7(7):e1002189. doi: 10.1371/journal.pgen.1002189 (PMC3141006; doi:10.1371/journal.pgen.1002189)
Supplement: Table S1 — List of mutant oriCII plasmids. (DOC) [file pgen.1002189.s006.doc]

**Table S1.** **Mutant *oriCII* plasmids**

| **Mutation** | **Isolates** | **Notes** |
| --- | --- | --- |
| **A. Deletions in *rctA* region** | | |
| ∆151-249 | A-11 |  |
| ∆112-252 | A-22, A-39, A-48, A-51 |  |
| ∆194-670 | A-15 |  |
| **B. Amino acid substitution** | | |
| D172Y | A-20 |  |
| T179I | A-61 |  |
| R195L | A-2 |  |
| T258K | A-26, A-47 |  |
| R269S | A-1 | Duigou et al., 2008 [7] |
| G270C | A-6, A-9, A-49 |  |
| G270V | A-21, A-55 |  |
| S274N | A-4 |  |
| D292Y | A-7, A-14, A-38 |  |
| S313R | A-10 |  |
| R321C | A-40 |  |
| R321G | A-25 |  |
| R321H | A-35 |  |
| R321S | A-54 |  |
| P330L | A-29 |  |
| P351L | A-19 |  |
| L365I | A-52 | Yamaichi et al., 2009 [21] |
| L357R | A-8 |  |
| Q373K | A-17 |  |
| A406D | A-31 |  |
| R413L | A-12, A-42, A-43 |  |
| V469L | A-59 |  |
| P516Q | A-57 | Yamaichi et al., 2009 [21] |
| E545L | A-27 |  |
| A558E | A-18 |  |
| A629E | A-37 |  |
| **C. Deletion in C-terminus [∆deletions(# of amino acid insertion, shown in Notes)]** | | |
| ∆618-658(12) | A-13 | DRKIDRRFSGTP* |
| ∆600-658(0) | A-32 | * |
| ∆595-658(2) | A-58 | TM* |
| ∆588-658(0) | A-36 | * |
| ∆574-658(8) | A-53 | LHQSKKSV* |
| ∆554-658(7) | A-5 | RSLTKTD* |
| ∆545-658(0) | A-45, A-46 | * |
| ∆543-658(16) | A-3 | VMKSPLLYRVIPLQKR* |
| ∆529-658(3) | A-50 | VGA* |
| ∆527-658(1) | A-24 | A* |
| ∆519-658(3) | A-60 | SLR* |
| ∆516-658(6) | A-28 | RIGTSE* |
| ∆508-658(2) | A-41 | RE* |
| ∆507-658(0) | A-30 | * |
| ∆506-658(58) | A-16 | SLSKLLELYNSQNESSKHLSIEKLIAGLAVRRKVCKLVQDGH IDETVYRALDEMAAGA* |
| ∆505-658(7) | A-44 | VIEHHRW* |
| ∆500-658(0) | A-56 | * |
| ∆553-631 | A-33 |  |
| ∆566-568 | A-34 |  |
