# Supplementary material for: Regulatory Cross-Talk Links Vibrio cholerae Chromosome II Replication and Segregation
Source: PLoS Genet. 2011 Jul 21;7(7):e1002189. doi: 10.1371/journal.pgen.1002189 (PMC3141006; doi:10.1371/journal.pgen.1002189)
Supplement: Table S2 — List of plasmids used for bacterial two hybrid assay. (DOC) [file pgen.1002189.s007.doc]

**Table S2. Plasmids used for bacterial two hybrid assay**

| **Name** | **Description** | **Resistance** | **Reference** |
| --- | --- | --- | --- |
| pKNT25 | p15A ori T25 | Km | Euromedex |
| pKT25 | p15A ori T25 | Km | Euromedex |
| pUT18 | pUC ori T18 | Amp | Euromedex |
| pUT18C | pUC ori T18 | Amp | Euromedex |
| pK rctB-T25 | p15A ori *rctB-*T25 fusion | Km | This study |
| pK T25-parB2 | p15A ori T25-*parB2* fusion | Km | This study |
| pK parA2-T25 | p15A ori parA2-T25 fusion | Km | This study |
| pU rctB-T18 | pUC ori *rctB-*T18 fusion | Amp | This study |
| pU T18-parB2 | pUC ori T18-*parB2* fusion | Amp | This study |
| pU parA2-T18 | pUC ori *parB2-*T18 fusion | Amp | This study |

Resistance; Amp, ampicillin; Km, kanamycin
